# Socioeconomic status, stressful life situations and mental health problems in children and adolescents: Results of the German BELLA cohort-study

**DOI:** 10.1371/journal.pone.0213700

**Published:** 2019-03-13

**Authors:** Franziska Reiss, Ann-Katrin Meyrose, Christiane Otto, Thomas Lampert, Fionna Klasen, Ulrike Ravens-Sieberer

**Affiliations:** 1 Department of Child and Adolescent Psychiatry, Psychotherapy, and Psychosomatics, University Medical Center Hamburg-Eppendorf, Hamburg, Germany; 2 Department of Epidemiology and Health Monitoring, Robert Koch-Institute, Berlin, Germany; Chiba Daigaku, JAPAN

## Abstract

**Aim:**

Children and adolescents with low socioeconomic status (SES) suffer from mental health problems more often than their peers with high SES. The aim of the current study was to investigate the direct and interactive association between commonly used indicators of SES and the exposure to stressful life situations in relation to children’s mental health problems.

**Methods:**

The prospective BELLA cohort study is the mental health module of the representative, population-based German National Health Interview and Examination Survey for children and adolescents (KiGGS). Sample data include 2,111 participants (aged 7–17 years at baseline) from the first three measurement points (2003–2006, 2004–2007 and 2005–2008). Hierarchical multiple linear regression models were conducted to analyze associations among the SES indicators household income, parental education and parental unemployment (assessed at baseline), number of stressful life situations (e.g., parental accident, mental illness or severe financial crises; 1- and 2-year follow-ups) and parent-reported mental health problems (Strength and Difficulties Questionnaire; 2-year follow-up).

**Results:**

All indicators of SES separately predicted mental health problems in children and adolescents at the 2-year follow-up. Stressful life situations (between baseline and 2-year follow-up) and the interaction of parental education and the number of stressful life situations remained significant in predicting children’s mental health problems after adjustment for control variables. Thereby, children with higher educated parents showed fewer mental health problems in a stressful life situation. No moderating effect was found for household income and parental employment. Overall, the detected effect sizes were small. Mental health problems at baseline were the best predictor for mental health problems two years later.

**Conclusions:**

Children and adolescents with a low SES suffer from multiple stressful life situations and are exposed to a higher risk of developing mental health problems. The findings suggest that the reduction of socioeconomic inequalities and interventions for families with low parental education might help to reduce children’s mental health problems.

## Introduction

Socioeconomic inequalities are an important topic in politics, social sciences and public health research. Families with a low socioeconomic status (SES) are deprived in multiple ways and suffer from a higher number of stressors related to finances, social relations, employment situations and health complaints than those with a high SES [1, 2]. These socioeconomic inequalities affect not only parents’ but also children’s lives. For instance, children with low SES often have worse access to education and social participation than their peers with high SES [3]. Moreover, children with low SES suffer more often from health problems than children with high SES [4]. Results from a time-series analysis of 34 countries from 2002 to 2010 showed that inequalities between socioeconomic groups increased in many domains of adolescent health; thereby, adolescents with a low SES are more affected by psychological and physical symptoms [5].

Worldwide, it is estimated that 13% to 20% of children and adolescents suffer from disabling mental illness [6, 7]. When symptoms of mental health problems occur early in life this has been shown to increase the risk of mental health problems in adulthood [8, 9].

Children and adolescents with low SES are two to three times more likely to develop mental health problems than their peers with high SES [10]. In numerous studies, indicators of low SES (commonly measured by the household income per capita, parental education and parental occupation status) were directly associated with increased mental health problems in children and adolescents [11–13]. Indicators of childhood SES differentiate in predicting the onset, persistence, and severity of mental disorders [14]. Household income and parental education have a stronger impact on the mental health problems of children and adolescents than parental unemployment or low occupation status, which refers to a low position in the occupational hierarchy [10]. Furthermore, parents with a university degree are more likely to have children with higher positive psychological health than children of parents with no university degree [15].

Additionally, low SES relates to a higher burden in different areas of everyday life and an exposure to stressful life situations. Studies concluded that negative life events and other stressors are clearly related to socioeconomic position [16] and lower parental education and lower household income were associated with higher stress levels irrespective of adolescent’s gender [17]. In more detail, SES is associated with the frequency of stressful life events and stress responses [18]. Furthermore, the exposure to negative life events and family stress partly explained the association between SES and the symptoms of mental health problems in a Swedish sample of adolescents [19]. This is in line with results of a longitudinal study by Koechlin and colleagues (2018) reporting that both childhood stressful life events and lower maternal education level significantly predicted adjustment problems in adolescence [20]. Similar findings were reported for the mediating role of life stressors on the relationship between SES and mental health status in young adults participating in a longitudinal US study [21]. Altogether, it can be assumed that low SES is associated with more problems and stressful life situations of the family, which increases the risk of children’s mental health problems. To date, studies investigating the combined effects of SES indicators and stressful life situations as well as their influence on mental health problems in children and adolescents are rare.

The objectives of our study were to investigate the direct and interactive effects of low SES (i.e., household income, parental education and parental unemployment) and stressful life situations in relation to mental health problems in children and adolescents aged 7 to 17 years at baseline. Blockwise multiple linear regression models were used to identify the direct effects of SES indicators (measured at baseline) and the number of stressful life situations (measured at 1- and 2-year follow-ups) on children’s mental health problems. The interactive effects of SES indicators and the number of stressful life situations with regard to children’s mental health problems were further examined. Additional risk factors for children’s mental health problems (e.g., family structure, initial mental health problems), along with age and gender were included in the analyses as control variables. The study uses data from a population-based representative sample of German children and adolescents from the BELLA cohort-study [22].

We focused on the following four hypotheses: i) all indicators of low SES (i.e., household income, parental education and parental unemployment) are separately associated with more mental health problems of children and adolescents at the 2-year follow-up, ii) a higher number of stressful life situations is associated with more mental health problems of children and adolescents at the 2-year follow-up, iii) the interaction of SES indicators with stressful life situations affects children’s and adolescents' mental health problems (moderation effect), and (iv) effects remain significant when control variables are added to the model.

## Materials and methods

### Study design

Analyses are based on the representative and prospective BELLA cohort study, which is the mental health module of the National Health Interview and Examination Survey for Children and Adolescents (KiGGS) in Germany [22]. The BELLA cohort study examines a randomly selected subsample of KiGGS. Potential study participants were chosen in a multistage random sampling from the official registers of the local residents’ registration offices, including 167 sample points throughout Germany. In the present study, data from the first three measurement points of the BELLA study were used: BELLA baseline assessment (2003–2006), 1-year follow-up (2004–2007) and 2-year follow-up (2005–2008). Where available, psychometrically sound and internationally tested measures were used to assess demographic characteristics, mental health problems and disorders in addition to risk and protective factors (e.g., a stressful life situation). Data were collected by computer-assisted telephone interviews and subsequent questionnaires. Parents provided written informed consent on behalf of their 7- to 17-year-old children. Adolescents aged 14 years or older gave their written informed consent. For all measurement points of the BELLA study, approvals from the ethics committee of the University Hospital Charité in Berlin and the Federal Commissioner for Data Protection in Germany were obtained. For further details on design and methods, see Ravens-Sieberer et al. [23].

### Participants

**Fig 1:**
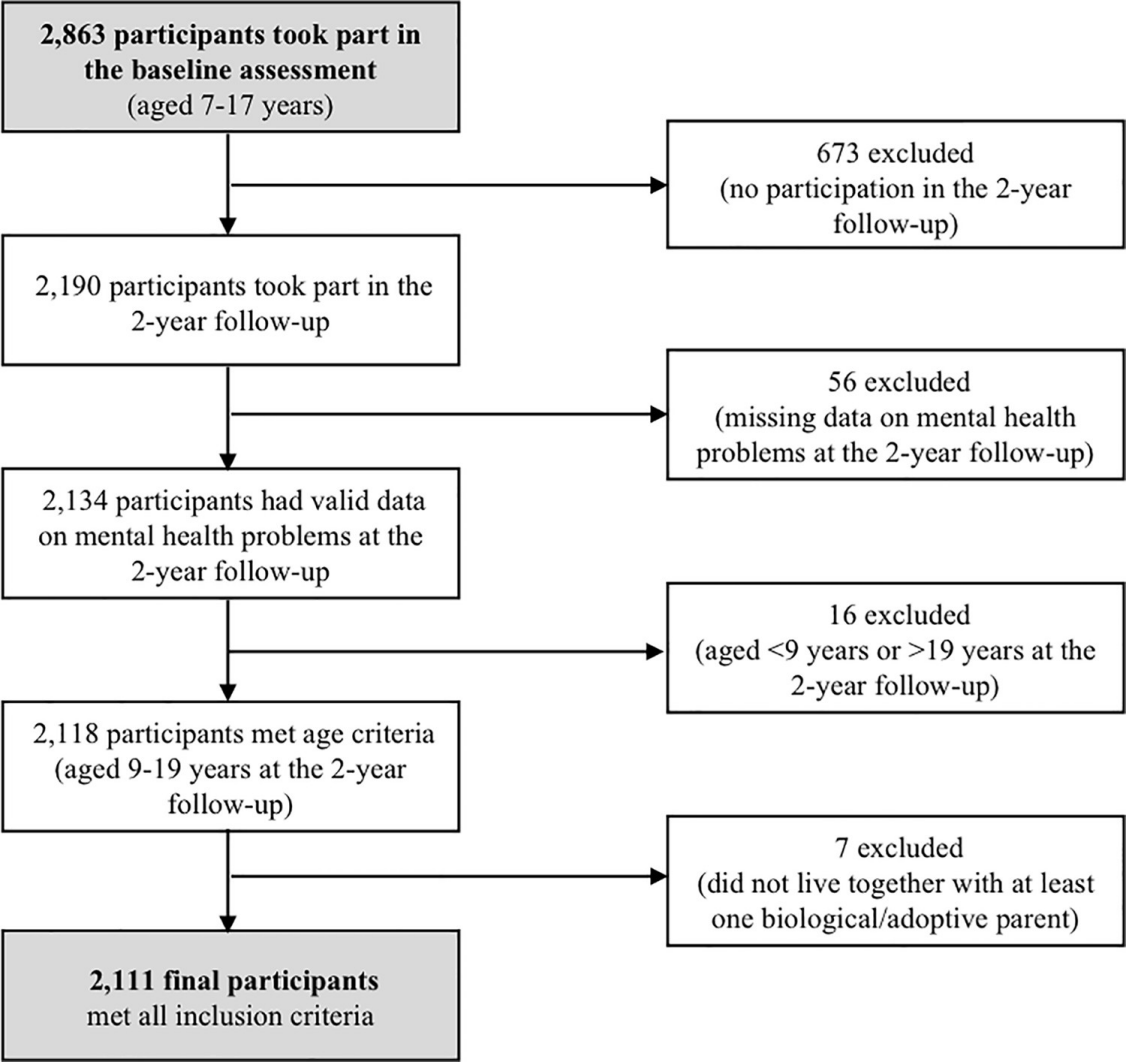
Flow chart for selection of study participants based on the inclusion criteria.

In total, a sample of 2,863 children, adolescents (aged 7 to 17 years) and their parents participated in the baseline assessment of the BELLA study. For the present study, longitudinal data collected over a period of two years were used (gathered at baseline, 1- and 2-year follow-ups). BELLA baseline participants were included in the present study if they i) participated in the 2-year follow-up (excluded: *n* = 673), ii) had valid data on mental health problems at the 2-year follow-up (excluded: *n* = 56 due to missing data in the Strengths and Difficulties Questionnaire), iii) meet age criteria (9 to 19 years) at the 2-year follow-up (excluded: *n* = 16 were younger than 9 years or older than 19 years), and iv) lived together with at least one biological parent or adoptive parent (excluded: *n* = 2 living with grandparents/other relatives, *n* = 3 living in a children home, *n* = 2 living on their own). Consequently, data from 2,111 children and adolescents could be analyzed. For a flow chart for selection of study participants based on inclusion criteria, see Fig 1.

### Measurements

#### Socioeconomic status

Parents provided information on the most commonly used *indicators of SES*: equivalent household net income (short: household income), parental education and parental occupation. The *equivalent household net income* was calculated by a family's approximate monthly net equivalent income adjusted for household size and age-specific needs of household members (Organization for Economic Cooperation and Development, OECD-modified equivalence scale: head of household = 1, additional adult household members = 0.5, children = 0.3) [24]. *Parental education* was measured by the mean of maternal and paternal years of education completed. Parents’ years of education were estimated by using categories of German school-leaving certificates (e.g., 13 years for German *Abitur* as the general qualification for university entrance; 10 years for German *Mittlere Reife*, roughly comparable to American high school diploma; zero years for people still enrolled in school). In addition, certificates of vocational qualifications were taken into account (e.g., 5 years for a university degree; 3 years for a completed vocational training; 1.5 years for a completed basic training, for example to become a parts processor). Thus, the highest educational degree (i.e., 18 years for a university degree) comprises the regular number of school years completed (i.e., 13 years) plus the average years of university education (i.e., 5 years) to achieve this educational attainment in Germany. The current *parental occupational status* referred to the employment status as whether at least one parent was unemployed.

#### Stressful life situation

A *stressful life situation* is defined by the level of stress caused by the occurrence of a certain life situation. In this study, the term “life situation” is preferred because the impact of a stressful life situation does not describe an event at a particular point in time but rather is seen as a process. At both measurement points (1- and 2-year follow-ups), parents were asked by means of a list of items if the following situations occurred over the past 12 months: 1) *own serious illness or accident*, 2) *own mental illness*, 3) *divorce or separation from a partner*, 4) *severe financial crisis*, 5) *loss of employment (respondent or partner)*, 6) *child problems in school* and 7) *trouble with the law or legal proceedings*. Items were offered with response options *no* (0) and *yes* (1). If the occurrence of a certain life situation was affirmed, parents were subsequently asked to rate their stress level caused by this situation on a 4-point scale (*not stressful* to *very stressful*). For the present analyses, responses to subsequent questions were dichotomized into *not or little stressful* (0) and *quite or very stressful* (1) and summed up to an overall score (ranging from 0 to 7) with higher scores indicating more stressful life situations. If certain life situations did not occur, it was included in the sum score as *not stressful (because not experienced)* (0). Finally, a sum score was calculated by gathering the overall scores for both measurement points and covering the additive number of stressful life situations over the investigated two years (ranging from 0 to 14 with higher scores indicating a higher number of stressful life situations in the family).

#### Mental health problems

*Mental health problems* in children and adolescents were assessed by the parent-reported Strengths and Difficulties Questionnaire (SDQ, [25]) at baseline and 2-year follow-up. The SDQ is a well-established, brief, reliable and valid screening questionnaire for mental health problems in children and adolescents [26]. For this study, the Total Difficulties Score was used to cover the four subscales of mental health problems (i.e., emotional symptoms, conduct problems, hyperactivity/inattention, and peer relationship problems) with 20 items and a range from 0 to 40. Higher scores indicated more severe mental health problems in children and adolescents. The items of the SDQ refer to the last 6 months and were answered on a three-point scale (*not true*, *somewhat true*, *certainly true*). In the current study, internal consistencies were Cronbach’s α = 0.71 and α = 0.72 for the baseline and 2-year follow-up, respectively.

#### Control variables (gender, age, family structure, and children’s mental health problems at baseline)

Age (in years), gender (0 = *female*, 1 = *male*) and mental health problems of children and adolescents as well as family structure were assessed at baseline as control variables. Children’s *mental health problems* were measured by the parent-reported Strengths and Difficulties Questionnaire [SDQ, 25]; for more detailed information, see paragraph above. *Family structure* was operationalized by children’s usual place of residence and dichotomized into *living with both biological parents* versus *not living with both biological parents*. The latter category included all children living in single-parent families (mother or father only), in step-parent families (mother or father with new partner) or living with adoptive parents. All control variables were included in the multiple linear regression analyses.

### Statistical analysis

Descriptive statistical analyses comprised the calculation of frequencies or means and standard deviations for all analyzed variables. Furthermore, a correlation matrix served to investigate bivariate associations between indicators of SES, number of stressful life situations, and mental health problems (baseline and 2-year follow-up). According to Cohen [27], we interpreted a correlation of *r* = .1 as *small*, *r* = .3 as *medium* and *r* = .5 as *large*. Multiple linear regression models were calculated using a hierarchical (blockwise) approach to test each of the four hypotheses with one model. Thus, children’s mental health problems (2-year follow-up) were predicted by:

Model 1: household income, parental education, parental unemployment (all assessed at baseline)—testing hypothesis (i),

Model 2: Model 1 plus number of stressful life situations (between baseline and 2-year follow-up)—testing hypothesis (ii),

Model 3: Model 2 plus interaction terms to test moderation effects (household income x number of stressful life situations, parental education x number of stressful life situations, parental unemployment x number of stressful life situations)—testing hypothesis (iii), and

Model 4: Model 3 plus control variables (gender, age, gender x age, family structure, mental health problems; all assessed at baseline)—testing hypothesis (iv).

For the regression analyses, the metric predictors household income and parental education as well as the control variable age were centered using the grand mean of the sample. Effect sizes, *p*-values and corresponding 95% confidence intervals (CI) are reported. The overall fit of the models was evaluated by adjusted *R^2^* statistics [28], and the significance of changes in model fit were determined by *R^2^*-Change and *F*-test [29]. To interpret the regression coefficients of the regression models (β), we used guidelines by Cohen [27]: β = .1 indicated a *small*, β = .3 a *medium* and β = .5 a *large* effect. Prior to model calculations, we replaced missing data of predictors and control variables using the Expectation-Maximization (EM) algorithm to include all cases (*N* = 2111). Missing values were below 2% for all predictors. In addition, a sensitivity analysis was computed to test the robustness of the results according to the missing imputation (results *with* vs. *without imputation*).

All analyses were computed using IBM SPSS Version 22. The significance level was determined as *p* < .05 for all analyses.

## Results

### Sample characteristics

In total, longitudinal data of *N* = 2,111 children and adolescents (48.7% female) were analyzed. At baseline, the participants were 7 to 17 years old (*M* = 11.96, *SD* = 3.09). Most children lived with both biological parents (78.4%), 11% of the children and adolescents lived with their mothers, 0.7% with their fathers or with their mother/father and a new partner (8.3% and 0.4%, respectively), and 0.9% with adoptive or foster parents. In most cases, the mother responded to questionnaires (baseline: 90.1%, 2-year follow-up: 90.7%).

Concerning the families’ SES, the equivalent household net income was 1,200 Euro, slightly below the average in Germany [30]. Parents had a mean education of 12.99 years of school and training (*SD* = 2.39), which corresponds to the average duration of school attainment in Germany (i.e., 12.65 years as determined in 2000 [31]). The years of education ranged from 1.5 to 18 years with 96.3% of parents having 10 to 18 years of education. In 12.1% of the families, at least one parent was unemployed; this finding is comparable to the unemployment rate in Germany (11.7% in 2005), which was published by the Federal Labour Office [32]. Further characteristics of the analyzed sample are presented in Table 1.

**Table 1:** Descriptive characteristics of the study population.

	Children and adolescents (*N* = 2,111)		
	*n*	Valid %	*M* (*SD*)
Gender	2,111		
*Male*	1,083	51.3	
*Female*	1,028	48.7	
Age (years)			
*Baseline (7–17 years)*	2,111		11.96 (3.09)
*2-year follow-up (9–19 years)*	2.111		14.09 (3.10)
Parental education (in years)	2,091		12.99 (2.39)
Household income (in 100€/month)	2,102		12.00 (5.82)
Parental unemployment	2,106		
*None*	1,851	87.7	
*At least one parent*	225	12.1	
Number of stressful life situations (counted between baseline and 2-year follow-up)	2,111		0.89 (1.43)
Family structure	2,107		
*Living with both biological parents*	1,655	78.4	
*Living without both biological parents*^1^	452	21.4	
SDQ total score			
*Baseline*	2,105		7.86 (5.11)
*2-year follow-up*	2,111		7.40 (5.10)

Note.

^1^, i.e., living in single-parent families, in step-parent families or with adoptive parents

SDQ = Strengths and Difficulties Questionnaire [Goodman, 1997].

In total, *n =* 897 (42.5%) of parents reported at least one stressful life situation between the baseline and 2-year follow-up. Within the measurement period of two years, the number of stressful life situations in the families ranged between zero and ten (*M* = 0.89, *SD* = 1.43). Most frequently, parents mentioned the following stressful life situations: *severe financial crisis* (*n* = 452, 10.7% of families), *child problems in school* (*n* = 442, 10.5%), and *serious illness or accident of a parent* (*n* = 330, 7.8%), for all frequencies see Table 2. Several stressful life situations were reported at both measurement points: for instance, a *severe financial crisis* (*n* = 121, 5.7%), *child problems in school* (*n* = 88, 0.2%) or *parental serious illness or accident* (*n* = 59, 2.8%). These life situations repeatedly occurred or seem to be long-lasting stressors for family life.

**Table 2:** Stressful life situations at all measurement points.

	Children and adolescents (N = 2.111)		
	*n* (valid %)		
**Stressful life situations^1^**	**T0-T1**	**T1-T2**	**Total^2^**
Parental serious illness or accident	145 (6.9)	185 (8.8)	330 (7.8)
Parental mental illness	100 (4.7)	95 (4.5)	195 (4.6)
Divorce or separation from partner	50 (2.4)	61 (2.9)	111 (2.6)
Severe financial crisis	217 (10.3)	235 (11.1)	452 (10.7)
Loss of employment (respondent or partner)	132 (6.3)	116 (5.5)	248 (5.9)
Child problems in school	209 (9.9)	233 (11.0)	442 (10.5)
Trouble with the law or legal proceedings	57 (2.7)	51 (2.4)	108 (2.6)

Note.

^1^multiple answers possible

T0 = Baseline, T1 = 1-year follow-up, T2 = 2-year follow-up

^2^total of stressful life situations between Baseline and 2-year follow-up.

### Bivariate analyses

The results of the bivariate analyses of household income, parental education, parental unemployment, number of stressful life situations and children’s mental health problems at baseline and 2-year follow-up are presented in Table 3. Bivariate correlation analyses revealed that a lower household income, lower parental education, and parental unemployment were associated with higher rates of mental health problems in children and adolescents at baseline and at the 2-year follow-up (Table 3). For household income and parental unemployment, effect sizes were significant but small, and for parental education, effect sizes were small to medium. Moreover, all three indicators of SES were significantly associated with the number of stressful life situations. In detail, families with lower household income (*r* = -.153; *p*≤.01), lower parental education (*r* = -.116; *p*≤.01), and parental unemployment (*r* = .163; *p*≤.01) reported more stressful life situations than families with high SES. Furthermore, more reported stressful life situations were significantly associated with higher rates of mental health problems in children and adolescents at the 2-year follow-up (*r* = .318; *p*≤.01). The mental health problems of children and adolescents measured at baseline were strongly related to mental health problems at the 2-year follow-up (*r* = .676, *p*≤.01) (see Table 3).

**Table 3:** Pairwise correlation coefficients of indicators of SES, number of stressful life situations, and mental health problems.

		Parental education (in years, T0, centred)	Unemployment of father and/or mother	Number of stressful life situations (counted, between T0 and T2)	SDQ, total score (T0, parent report)	SDQ, total score (T2, parent report)
Household income (in 100€, T0, centred)	*r*	.489**	-.286**	-.153**	-.169**	-.155**
	*n*	2,085	2,098	2,102	2,097	2,102
Parental education (in years, T0, centred)	*r*		-.147**	-.116**	-.197**	-.176**
	*n*		2,089	2,091	2,087	2,091
Parental unemployment (father and/or mother)	*r*			.163**	.126**	.106**
	*n*			2,106	2,102	2,106
Number of stressful life situations (counted, between T0 and T2)	*r*				.249**	.318**
	*n*				2,105	2,111
SDQ, total score (T0, parent report)	*r*					.676**
	*n*					2,105

*Note*. T0 = Baseline, T2 = 2-year follow-up, SDQ = Strengths and Difficulties Questionnaire [Goodman, 1997], significant effects in bold.

* p ≤.05

** p ≤ .01.

### Multiple linear regression

The results of the hierarchical multiple linear regression are presented in Table 4. Findings by means of Model 1 (adjusted *R^2^* = .04) indicated that higher household income, higher parental education and parental employment are significantly associated with lower mental health problems in children and adolescents at the 2-year follow-up; the corresponding effect sizes were small according to Cohen [27] and slightly stronger for parental education (*β* = -.13; *p* < .001) than for household income (*β* = -.07; *p* = .004) and parental unemployment (*β* = 0.07; *p* = .003).

**Table 4:** Relationship between indicators of SES, number of stressful life situations and its interaction on mental health problems in children and adolescents two years later.

	Model 1				Model 2				Model 3				Model 4			
	*B*	β	*p*	95% CI of *B*	*B*	β	*p*	95% CI of *B*	*B*	β	*p*	95% CI of *B*	*B*	β	*p*	95% CI of *B*
Intercept	7.27		< .001	7.04;7.50	6.41		< .001	6.14;6.64	6.38		< .001	6.13;6.61	1.90		< .001	1.58;2.23
Household income (in 100€, T0, centered)	-0.06	**-.07**	.004	-0.11;-0.02	-0.04	-.05	.054	-0.08;-0.00	-0.04	-.05	.093	-0.09;0.01	0.00	.00	.910	-0.03;0.04
Parental education (in years, T0, centered)	-0.28	**-.13**	< .001	-0.39;-0.18	-0.25	**-.12**	< .001	-0.34;-0.14	-0.16	**-.08**	.005	-0.28;-0.04	-0.00	-.00	.973	-0.09;0.09
Parental unemployment (father and/or mother)	1.02	**.07**	.003	0.34;1.70	0.43	.03	.199	-0.34;0.99	0.91	**.06**	.033	0.07;1.74	0.19	.01	.550	-0.44;0.83
Number of stressful life situations (counted, between T0 and T2)					1.04	**.29**	< .001	0.91;1.20	1.04	**.29**	< .001	0.88;1.21	0.54	**.15**	< .001	0.41;0.67
Household income × number of stressful life situations									-0.01	-.01	.746	-0.04;0.03	-0.01	-.02	.371	-0.04;0.01
Parental education × number of stressful life situations									-0.09	**-.08**	.003	-0.16;0.03	-0.09	**-.07**	< .001	-0.13;-0.04
Parental unemployment × number of stressful life situations									-0.39	**-.06**	.049	-0.77;-0.00	-0.24	-.04	.110	-0.53;0.05
Gender of child (male)													0.16	.02	.326	-0.16;0.47
Age of child (in years, T0, centered)													-0.11	**-.07**	.002	-0.18;-0.04
Gender × age of child (T0)													-0.15	**-.07**	.003	-0.25;-0.05
Family structure: without both biological parents (T0)													0.39	.03	.053	-0.01;0.79
SDQ, total score (T0, parent report)													0.61	**.61**	< .001	0.58;0.64
Model fit indices																
Adjusted *R*^2^	.04				.12				.13				.50			
Δ*F*(*df*_1_,*df*_2_), *p*-value	Δ*F*(3, 2107) = 30.76, *p* < .001				Δ*F*(1, 2106) = 197.66, *p* < .001				Δ*F*(3, 2103) = 4.19, *p* = .006				Δ*F*(5, 2098) = 309.81, *p* < .001			

*Note*. Model 1: effects of household income, parental education, parental unemployment (all assessed at baseline) on mental health problems in children and adolescents two years later–testing hypothesis 1; Model 2: Model 1 plus the number of stressful life situations (between baseline and 2-year follow-up)–testing hypothesis 2; Model 3: Model 2 plus interaction terms to test moderation effects (household income x number of stressful life situations, parental education x number of stressful life situations, parental unemployment x number of stressful life situations)–testing hypothesis 3; Model 4: Model 3 plus control variables (gender, age, gender x age, family structure, mental health problems; all assessed at baseline)–testing hypothesis 4.

T0 = Baseline assessment, T2 = 2-year follow-up, SDQ = Strengths and Difficulties Questionnaire [Goodman, 1997], significant effects in bold.

Findings by means of Model 2 (adjusted *R^2^* = .12) indicated that the number of stressful life situations contributed significantly to children’s mental health problems at the 2-year follow-up. More stressful life situations indicated higher rates of children’s mental health problems (medium effect; *β* = 0.29; *p*≤.001). In this model, parental education was still associated with children’s mental health problems, whereas household income and parental unemployment had no significant effects on children’s mental health problems at the 2-year follow-up.

In Model 3 (adjusted *R^2^* = .13), interaction terms of the independent variables were added to the previous predictors to investigate moderation effects. For the interaction of parental education and the number of stressful life situations, a significant (but small) effect on children’s mental health problems was found at the 2-year follow-up (*β* = -0.08; *p* = .003). Thus, children of parents with higher education living in a stressful life situation showed fewer mental health problems than children of parents with lower education living in a stressful life situation. Moreover, parental employment status also moderated the association between the number of stressful life situations and children’s mental health problems significantly (*β* = -0.06, *p* = .049). Thus, parental unemployment increases the risk for mental health problems in children and adolescents in general (main effect) and especially when less stressful life situations were reported (interaction effect). In families with a high number of stressful life situations, parental employment status has no additional negative effect on children’s and adolescents’ mental health.

Finally, Model 4 (adjusted *R^2^* = .50) included the control variables age, gender, family structure and children’s mental health problems at baseline (in addition to previous predictors and interaction terms). In this model, none of the single indicators of SES remained statistically significant; however, the number of stressful life situations continued to be a significant predictor of children’s mental health problems at the 2-year follow-up. Overall, the results of the moderator analyses (including the control variables) revealed that children are at higher risk of showing mental health problems if their parents have lower education and report a higher number of stressful life situations than their peers with a high number of stressful life situations but higher-educated parents. Therefore, the number of stressful life situations can be attenuated by a higher level of parental education. Our findings revealed the importance of parental education, but neither household income nor parental unemployment had significant effects on mental health in children and adolescents at the 2-year follow-up in the final model (Model 4).

The inclusion of control variables (i.e., age, gender, family structure and children’s mental health problems at baseline) in Model 4 indicated that children’s mental health problems at baseline were the strongest predictor for their mental health problems at the 2-year follow-up (*β* = 0.61; *p*≤.001). Moreover, the age of the participants significantly predicted children’s mental health problems, with younger children showing more noticeable problems than older children. Furthermore, a significant interaction of age and gender was observed: boys had a stronger decrease in mental health problems over time than girls. Living without both biological parents was associated with higher mental health problems at the 2-year follow-up, but this effect did not reach significance (*p* = .053). Overall, 50% of the variance in children’s mental health problems at the 2-year follow up could be explained in the final model.

To test the robustness of the presented results, we compared the models with and without missing data imputation (statistics not presented). This sensitivity analysis confirmed our results, indicating similar coefficients, significances and proportions of explained variance.

## Discussion

The present study was the first to investigate the direct and interactive association between single indicators of SES and stressful life situations in relation to mental health problems in children and adolescents using data from a large population-based sample from Germany. All indicators of low SES as well as a high number of stressful life situations were associated with more mental health problems in children and adolescents. As a main finding of the study, only number of stressful life situations and the interaction between parental education and number of stressful life situations remained significant in predicting children’s mental health problems at the 2-year follow-up after adjustment for fundamental variables. Nonetheless, existing children’s mental health problems at baseline was the strongest predictor of mental health problems at the 2-year follow-up.

In more detail, the study revealed that each indicator of SES separately contributed to children’s mental health problems at the 2-year follow-up; however, the detected effects were small for household income and parental unemployment, and small to medium for parental education. Thus, parental education was the strongest predictor, whereby children from families with higher-educated parents showed a lower risk of developing mental health problems than their peers with lower-educated parents. The importance of parental education within the indicators of SES was also determined by other studies [33]. McLaughlin et al. [14] reported in a US nationally representative sample of 5,692 adults that low parental education, although unrelated to disorder onset, significantly predicted disorder persistence and severity, whereas financial hardship predicted the onset of disorders at every life-course stage but showed no relation with disorder persistence or severity. Parental occupation had no significant impact on the onset, persistence and severity of mental disorders [14]. Our results are in line with previous results of the BELLA study investigating trajectories of mental health problems by maternal education: Children of mothers with low education had significantly more mental health problems during childhood and adolescence than children of mothers with high education [34]. Therefore, education not only affects income and occupational success but also helps people make better decisions about health, marriage, parenting and improves social interaction [35]. All of these skills are important in addressing the mental health problems of children and adolescents.

The effects of single SES indicators on children’s mental health problems (Model 1) partly disappeared when further variables were included (see Models 2 to 4). The results revealed that SES indicators explain the occurrence of mental health problems in children and adolescents only to some extent and must thus be considered in the context of other influencing circumstances. Families with low SES are exposed to multiple mechanisms of social segregation and disadvantage [16]. The accumulation of stressors or negative life situations is linked to these mechanisms. The great advance of this study was to observe the impact of a stressful life situation within the period of two years and therefore covered a relatively wide but clearly defined timespan.

Our results indicated that the number of stressful life situations, such as parental mental illness or accident, a severe financial crisis, loss of employment, child’s school problems, divorce or separation or trouble with the law, are more likely in families with low SES than in those with high SES. Furthermore, our study findings supported the second hypothesis that a higher number of stressful life situations is associated with more mental health problems in children and adolescents at the 2-year follow-up (Model 2). A Norwegian study found comparable results, whereby the accumulation of negative life events and the presence of family stressors partly explained the relation between mental health symptoms and SES in children and adolescents aged 11 to 13 years [19]. A National Epidemic Survey from the US with more than 30,000 participants aged 18 to 24 years reported similar results, whereby exposure to a number of stressful life events was examined as an important pathway through which SES and other demographic variables impact mental health in young adults [21]. Our study contributes findings to this research field, indicating that these associations are already visible in young children. Previous findings of the BELLA study also showed that mental health problems were more likely to occur between the ages of 7 and 12 and after the age of 19 years [23] and highlights the importance of including younger children in the examination.

Moreover, the pathway of stressful life situations through which SES impacts mental health is also recognizable in intergenerational relations between parents and their children. SES-associated stressful life situations during childhood and adolescence have long-term effects, as results from a French longitudinal study suggest that the experienced accumulation of negative childhood situations not only contributes to children’s current mental health problems, such as depression or anxiety, but also continues to affect their mental health in adulthood [36]. The findings of a review concluded that differential exposure to stress and negative life events are one of the mechanisms in which socioeconomic inequalities in health are produced in society [16]. Therefore, low SES and the experience of stressful life situations are mutually associated with each other and can therefore affect each other. Intergenerational mobility, i.e., the possibility of changing an individual’s social position compared to parental social position is linked to health inequalities, indicating that social advancement has a positive effect on health, whereas social decline has a negative effect on health [37].

Finally, our study findings partly supported the third hypothesis because of the interaction of one SES indicator, i.e., parental education, and the number of stressful life situations, which affected children’s and adolescents' mental health problems. Household income and parental unemployment showed no moderation effects on the association between a stressful life situation and children’s mental health problems (Model 4). Therefore, the effect of a stressful life situation on children’s mental health problems depends on the level of parental education: children of higher-educated parents are less affected by a stressful life situation and for that reason less likely to develop mental health problems than their peers with lower-educated parents. Thus, parental education can be interpreted as a major resource to avoid the development of children’s mental health problems, even if families suffer from stressful life situations. Possibly, higher educated parents experience life situations less stressful compared to less educated parents and/or are better equipped to handle stressful life situations. Grzywacz and colleagues (2004) found in a cross-sectional analysis a stronger negative impact of daily stressors on mental health among less educated adults; even if higher-educated adults reported more daily stressors, stressors reported by those with less education were more severe [38]. Additionally, women with higher education described lower perceived stress and greater control experiences in everyday life [39] and high education was found to be an important sociodemographic factor of various coping strategies [40]. Individuals with a higher level of education have more cognitive abilities and a better social position, which also buffers the impact of a stressful life situation on psychological distress [41]. A high parental education can be considered as one social determinant that provides the knowledge to deal with stressful life situations. With regard to the common measurements of SES, we assume that the strong impact of parental education can be partly explained as SES indicators built on one another. Concerning intragenerational mobility, educational attainment is an essential aspect of occupational success and financial resources [42].

Furthermore, our final model (Model 4) indicated that existing mental health problems in children and adolescents at baseline were the strongest predictor of mental health problems two years later. The results highlight the importance of persistence and early onset of mental health problems in childhood. A previous finding of the BELLA study showed that over a 6-year period, 10.2% of all children showed persistent, acute or recurrent mental health problems [23]. Moreover, mental health problems in childhood often persist until adulthood. Findings from the US National Comorbidity Survey stated that half of all lifetime cases start by the age of 14 [43]. Overall, our study findings underline the focus on longitudinal analyses because mental health problems in children are a critical issue in this sensitive phase of development from childhood to adolescence and further on to young adulthood.

### Strengths

The BELLA study is one of the most important cohort-studies that examines mental health problems in a population-based representative sample of children, adolescents and young adults in Germany. The strengths of the study are the large sample size and its longitudinal design, which enables the examination of mental health problems over time, including children aged seven years or older. Our contribution to research involves the analysis of single indicators of SES, which allows a deeper consideration of the differences between the commonly used indictors of SES. The hierarchical theory-based modeling in linear regression analyses helped to understand the disappearing effect of SES indicators on mental health problems in children and adolescents. Our results highlight the importance of considering a wider spectrum of living circumstances, e.g., health complaints, schooling, or dealing with difficult situations, in families with a low SES in future research. Finally, examination of the number of stressful life situations that occurred between different measuring points significantly contributes to a better understanding of the association between low SES and mental health problems in children and adolescents. This study takes the temporality of these situations into account and is therefore not limited to a cross-sectional time point.

### Limitations

Despite the strengths of this study, some limitations should be considered. First, indicators of SES were measured only by parent-reports at baseline. No data were available to consider changes in SES at the follow-up measurement points. Nonetheless, SES indicators such as parental education are supposed to be relatively stable in this age group. Second, drop-outs within the cohort of the BELLA study were more frequent for participants with low SES (2-year follow-up: OR = 1.06; 95% CI = 1.02–1.10) but independent of parent-reported general health or mental health of children and adolescents as reported by Ravens-Sieberer et al. [23]. Third, because we included young children from age seven or older in our analyses, mental health problems in children and adolescents were gathered by parent-reports.

### Conclusion

In conclusion, the impact of a stressful life situation on mental health problems in children and adolescents depends on the SES. Children from families with low SES are at higher risk of suffering from different stressful life situations. Furthermore, a stressful life situation is associated with mental health problems in children and adolescents. For this reason, it is important to focus not only on the indicators of SES, such as household income, parental education or parental occupation, but also on the broader current life situation with various burdens of stress in analyses on the mental health of children and adolescents. For future research, it would be interesting to examine other indicators (besides SES) that affect the association between a stressful life situation and children’s mental health, e.g., personal and social resources (e.g., social support or self-efficiency). In terms of opportunities for intervention and prevention, the aspect of parental education turned out as the most critical issue. Children with less educated parents obviously need more support in dealing with stressful life situations (e.g., parental illness or accident or severe financial crises) than their peers in a comparable situation but with higher-educated parents.

## Supporting information

S1 Data Socioeconomic status, stressful life situations and mental health. (XLS)

## References

[1] Senn TE, Walsh JL, Carey MP. The Mediating Roles of Perceived Stress and Health Behaviors in the Relation Between Objective, Subjective, and Neighborhood Socioeconomic Status and Perceived Health. Ann Behav Med. 2014;48(2): 215–24. 10.1007/s12160-014-9591-1 24648016 PMC4156915

[2] Weyers S, Dragano N, Mobus S, Beck EM, Stang A, Mohlenkamp S, et al Poor social relations and adverse health behaviour: stronger associations in low socioeconomic groups? Int J Public Health. 2010;55(1): 17–23. 10.1007/s00038-009-0070-6 19774341

[3] Engels D, Thielebein C. Zusammenhang von sozialer Schicht und Teilnahme an Kultur-, Bildungs- und Freizeitangeboten für Kinder und Jugendliche. [Association between social class and participation in cultural, education and leisure programs for children and adolescents.]. Köln; 2011.

[4] Vukojevic M, Zovko A, Talic I, Tanovic M, Resic B, Vrdoljak I, et al Parental Socioeconomic Status as a Predictor of Physical and Mental Health Outcomes in Children—Literature Review. Acta Clin Croat. 2017;56(4): 742–8. 10.20471/acc.2017.56.04.23 29590731

[5] Elgar FJ, Pfortner TK, Moor I, De Clercq B, Stevens GW, Currie C. Socioeconomic inequalities in adolescent health 2002–2010: a time-series analysis of 34 countries participating in the Health Behaviour in School-aged Children study. Lancet. 2015;385(9982): 2088–95. 10.1016/S0140-6736(14)61460-4 25659283

[6] Polanczyk GV, Salum GA, Sugaya LS, Caye A, Rohde LA. Annual research review: A meta-analysis of the worldwide prevalence of mental disorders in children and adolescents. J Child Psychol Psychiatry. 2015;56(3): 345–65. 10.1111/jcpp.12381 25649325

[7] Belfer ML. Child and adolescent mental disorders: the magnitude of the problem across the globe. J Child Psychol Psychiatry. 2008;49(3): 226–36. 10.1111/j.1469-7610.2007.01855.x 18221350

[8] Sv Stumm, Deary IJ, Kivimäki M, Jokela M, Clark H, Batty GD. Childhood behavior problems and health at midlife: 35-year follow-up of a Scottish birth cohort. J Child Psychol Psychiatry. 2011;52(9): 992–1001. 10.1111/j.1469-7610.2011.02373.x 21294730

[9] Roza SJ, Hofstra MB, van der Ende J, Verhulst FC. Stable prediction of mood and anxiety disorders based on behavioral and emotional problems in childhood: a 14-year follow-up during childhood, adolescence, and young adulthood. Am J Psychiatry. 2003;160(12): 2116–21. 10.1176/appi.ajp.160.12.2116 14638580

[10] Reiss F. Socioeconomic inequalities and mental health problems in children and adolescents: A systematic review. Soc Sci Med. 2013;90: 24–31. 10.1016/j.socscimed.2013.04.026 23746605

[11] Najman JM, Hayatbakhsh MR, Clavarino A, Bor W, O'Callaghan MJ, Williams GM. Family poverty over the early life course and recurrent adolescent and young adult anxiety and depression: a longitudinal study. Am J Public Health. 2010;100(9): 1719–23. 10.2105/AJPH.2009.180943 20634459 PMC2920957

[12] Amone-P'Olak K, Burger H, Ormel J, Huisman M, Verhulst FC, Oldehinkel AJ. Socioeconomic position and mental health problems in pre- and early-adolescents: the TRAILS study. Social Psychiatry Psychiatric Epidemiology. 2009;44(3): 231–8. 10.1007/s00127-008-0424-z 18714424

[13] Ravens-Sieberer U, Erhart M, Gosch A, Wille N. Mental health of children and adolescents in 12 European countries-results from the European KIDSCREEN study. Clin Psychol Psychother. 2008;15(3): 154–63. 10.1002/cpp.574 19115436

[14] McLaughlin KA, Breslau J, Green JG, Lakoma MD, Sampson NA, Zaslavsky AM, et al Childhood socio-economic status and the onset, persistence, and severity of DSM-IV mental disorders in a US national sample. Soc Sci Med. 2011;73(7): 1088–96. 10.1016/j.socscimed.2011.06.011 21820781 PMC3191493

[15] Padilla-Moledo C, Ruiz JR, Castro-Pinero J. Parental educational level and psychological positive health and health complaints in Spanish children and adolescents. Child Care Health Dev. 2016;42(4): 534–43. 10.1111/cch.12342 27097753

[16] Lantz PM, House JS, Mero RP, Williams DR. Stress, life events, and socioeconomic disparities in health: Results from the Americans' changing lives study. J Health Soc Behav. 2005;46(3): 274–88. 10.1177/002214650504600305 16259149

[17] Glasscock DJ, Andersen JH, Labriola M, Rasmussen K, Hansen CD. Can negative life events and coping style help explain socioeconomic differences in perceived stress among adolescents? A cross-sectional study based on the West Jutland cohort study. BMC Public Health. 2013;13: 532 10.1186/1471-2458-13-532 23724872 PMC3679909

[18] Baum A, Garofalo JP, Yali AM. Socioeconomic status and chronic stress—Does stress account for SES effects on health? In: Adler NE, Marmot M, McEwen B, Stewart J, editors. Socioeconomic Status and Health in Industrial Nations: Social, Psychological, and Biological Pathways. Annals of the New York Academy of Sciences. 896 New York: New York Acad Sciences; 1999 p. 131–44. 10.1111/j.1749-6632.1999.tb08111.x 10681894

[19] Boe T, Serlachius AS, Sivertsen B, Petrie KJ, Hysing M. Cumulative effects of negative life events and family stress on children's mental health: the Bergen Child Study. Soc Psychiatry Psychiatr Epidemiol. 2018;53(1): 1–9. 10.1007/s00127-017-1451-4 29090324

[20] Koechlin H, Donado C, Berde CB, Kossowsky J. Effects of Childhood Life Events on Adjustment Problems in Adolescence: A Longitudinal Study. J Dev Behav Pediatr. 2018;39(8): 629–41. 10.1097/DBP.0000000000000596 29944491

[21] Businelle MS, Mills BA, Chartier KG, Kendzor DE, Reingle JM, Shuval K. Do stressful events account for the link between socioeconomic status and mental health? J Public Health. 2014;36(2): 205–12. 10.1093/pubmed/fdt060 PMC4041099 23764393

[22] Ravens-Sieberer U, Kurth BM. The mental health module (BELLA study) within the German Health Interview and Examination Survey of Children and Adolescents (KiGGS): study design and methods. Eur Child Adolesc Psychiatry. 2008;17(1): 10–21. 19132300 10.1007/s00787-008-1002-3

[23] Ravens-Sieberer U, Otto C, Kriston L, Rothenberger A, Döpfner M, Herpertz-Dahlmann B, et al The longitudinal BELLA study: Design, methods and first results on the course of mental health problems. Eur Child Adolesc Psychiatry. 2015;24(6): 651–63. 10.1007/s00787-014-0638-4 25428179

[24] Lampert T, Müters S, Stolzenberg H, Kroll L, Group KS. Messung des sozioökonomischen Status in der KiGGS-Studie–Erste KiGGS-Folgebefragung (KiGGS Welle 1). [Measurement of socioeconomic status in the KiGGS study. First follow-up (KiGGS Wave 1).]. Bundesgesundheitsblatt—Gesundheitsforschung—Gesundheitsschutz 2014;57(7). 10.1007/s00103-014-1974-8 24950825

[25] Goodman R. The Strengths and Difficulties Questionnaire: a research note. J Child Psychol Psychiatry. 1997;38(5): 581–6. 9255702 10.1111/j.1469-7610.1997.tb01545.x

[26] Goodman R. Psychometric properties of the strengths and difficulties questionnaire. J Am Acad Child Adolesc. 2001;40(11):1337–45. 10.1097/00004583-200111000-00015 11699809

[27] Cohen J. Statistical power analysis for the behavioral sciences 2ed Hillsdale, NJ: Lawrence Erlbaum Associates; 1988.

[28] Nagelkerke NJD. A note on a general definition of the coefficient of determination. Biometrika. 1991;78(3): 691–2.

[29] Field A. FiDiscovering statistics using IBM SPSS statistics.: SAGE Publications; 2013.

[30] Statistisches Bundesamt [German Federal Statistical Office]. Wirtschaftsrechnungen. Einkommens- und Verbrauchsstichprobe Einkommensverteilung in Deutschland [Economic accounts. Distribution of income and consumption in Germany.]. Wiesbaden: Statistischen Bundesamt; 2006.

[31] Baten J, de Jong H. Internationale Vergleiche [International comparisons] In: Rahlf T, editor. Deutschland in Daten Zeitreihen zur Historischen Statistik [Germany in facts Histrical statistics]. Bonn: Bundeszentrale für politische Bildung; 2015 p. 304–19.

[32] Bundesagentur für Arbeit [Federal Labour Office]. II.C. Arbeitslosigkeit [Unemployment] Nürnberg: Bundesagentur für Arbeit; 2006.

[33] Davis E, Sawyer MG, Lo SK, Priest N, Wake M. Socioeconomic risk factors for mental health problems in 4-5-year-old children: Australian population study. Acad Pediatr. 2010;10(1): 41–7. 10.1016/j.acap.2009.08.007 20129480

[34] Meyrose A-K, Klasen F, Otto C, Gniewosz G, Lampert T, Ravens-Sieberer U. Benefits of maternal education for mental health trajectories across childhood and adolescence. Soc Sci Med. 2018;202: 170–8. 10.1016/j.socscimed.2018.02.026 29554584

[35] Oreopoulos P, Salvanes KG. Priceless: The Nonpecuniary Benefits of Schooling. J Econ Perspect. 2011;25(1): 159–84.

[36] Melchior M, Touchette E, Prokofyeva E, Chollet A, Fombonne E, Elidemir G, et al Negative events in childhood predict trajectories of internalizing symptoms up to young adulthood: an 18-year longitudinal study. PloS one. 2014;9(12): e114526 10.1371/journal.pone.0114526 25485875 PMC4259330

[37] Gunther S, Moor I, Knochelmann A, Richter M. Intergenerationale Mobilität und gesundheitliche Ungleichheiten in Ost- undWestdeutschland. Eine Trendanalyse von 1992 bis 2012. [Intergenerational mobility and health inequalities in East and West Germany: A trend analysis from 1992 to 2012]. Bundesgesundheitsblatt, Gesundheitsforschung, Gesundheitsschutz. 2018;61(1):78–88. 10.1007/s00103-017-2655-1 29138900

[38] Grzywacz JG, Almeida DM, Neupert SD, Ettner SL. Socioeconomic status and health: a micro-level analysis of exposure and vulnerability to daily stressors. J Health Soc Behav. 2004;45(1): 1–16. 10.1177/002214650404500101 15179904

[39] Gallo LC, Shivpuri S, Gonzalez P, Fortmann AL, de los Monteros KE, Roesch SC, et al Socioeconomic status and stress in Mexican-American women: a multi-method perspective. J Behav Med 2013;36(4): 379–88. 10.1007/s10865-012-9432-2 22644814 PMC3929319

[40] Holahan CJ, Moos RH. Personal and contextual determinants of coping strategies. J Pers Soc Psychol. 1987;52(5):946–55. 3585703 10.1037//0022-3514.52.5.946

[41] Mandemakers JJ, Monden CWS. Does education buffer the impact of disability on psychological distress? Soc Sci Med (1982). 2010;71(2):288–97. 10.1016/j.socscimed.2010.04.004 20488601

[42] Pollak R, Allmendinger J, Trappmann M, Ehlert M, Gatermann D, Heisig J, et al Soziale Mobilität, Ursachen für Auf- und Abstiege. Studie für den 4 Armuts- und Reichtumsbericht der Bundesregierung im Auftrag des Bundesministeriums für Arbeit und Soziales Berlin; 2013.

[43] Kessler RC, Berglund P, Demler O, Jin R, Merikangas KR, Walters EE. Lifetime prevalence and age-of-onset distributions of DSM-IV disorders in the National Comorbidity Survey Replication. Arch Gen Psychiatry. 2005;62(6):593–602. 10.1001/archpsyc.62.6.593 15939837

